# Mind the G(ap): bridging prevention needs and approaches for GHB/GBL users and their social environment

**DOI:** 10.1186/s12954-025-01152-9

**Published:** 2025-01-10

**Authors:** Antonia Bendau, Lukas Roediger, Andrea Piest, Rüdiger Schmolke, Katharin Ahrend, Moritz Bruno Petzold, Twyla Michnevich, Felix Betzler

**Affiliations:** 1 Department of Psychiatry and Neurosciences, CCM, Charité – Universitätsmedizin Berlin, corporate member of Freie Universität Berlin and Humboldt Universität zu Berlin, Charitéplatz 1, 10117 Berlin, Germany; 2 Notdienst Berlin e.V, Berlin, Germany; 3 BISS – Bundesinitiative für Sexualisierten Substanzkonsum e.V., Munich, Germany; 4 University of Applied Sciences Potsdam, Potsdam, Germany; 5 Clubcommission – Netzwerk der Berliner Clubkultur e.V., Berlin, Germany; 6 Awareness Akademie, Berlin, Germany; 7 Department of Psychology, MSB Medical School Berlin, Berlin, Germany

**Keywords:** Gamma-hydroxybutyric acid, Gamma-butyrolactone, GHB, GBL, Substance use, Drugs, Harm reduction, Addiction, Psychoactive substance

## Abstract

**Background:**

Gamma-hydroxybutyrate (GHB) and its precursors gamma-butyrolactone (GBL) and 1,4-butanediol (BD) have become a significant concern due to the increase in their recreational use and the high risks associated with it (e.g., overdose, addiction, life-threatening withdrawal syndromes). However, targeted prevention and treatment strategies are lacking, and little is known about the specific needs of users regarding supportive approaches.

**Methods:**

To address this gap, a mixed-methods longitudinal study was conducted with two waves of online data collection (11/2022–01/2023; 11/2023–01/2024) in Germany. The adult convenience sample (*N* = 2,196, with *n* = 240 participating in the follow-up) was mostly connected to Berlin’s nightlife scene and included GHB/GBL/BD users and their (non-user) social environment. Perceptions and needs regarding prevention and harm-reduction, reasons and measures of decreasing use, and the impact of GHB/GBL/BD use were analyzed both quantitatively and qualitatively.

**Results:**

Education, harm reduction strategies, and specialized support options were welcomed by users and non-users, while restrictive approaches were viewed negatively, particularly by heavy users. Many participants expressed a desire to reduce GHB/GBL/BD use, driven primarily by health concerns, immediate use risks, and addiction, but only few participants had previously accessed preventive/therapeutic services. The follow-up showed little change in perceptions and experiences over time.

**Conclusions:**

The findings underscore the need for comprehensive and integrative prevention and treatment strategies for GHB/GBL/BD use, with harm reduction approaches prioritized over restrictions. They provide a crucial foundation for future research and interventions and emphasize the necessity of adequately addressing the growing issues related to GHB/GBL/BD use.

**Trial registration:**

The study protocol was pre-registered with the German registry for clinical studies (Deutsches Register Klinischer Studien; drks.de/search/de/trial/DRKS00030608) on October 28, 2022.

## Background

*Gamma-hydroxybutyrate* (GHB), along with its chemical precursors *gamma-butyrolactone* (GBL) and *1,4-butanediol* (1,4BD; BDO; BD), has emerged as a significant concern in recreational substance use [1]. Initially synthesized in the 1960s, GHB has been approved for limited medical uses (e.g. as an anesthetic and for the treatment of narcolepsy) and was formerly sometimes used for performance-enhancement in the bodybuilding scene [1, 2]. These applications soon became overshadowed by its growing popularity as a recreational drug. Since 2002, GHB has been classified under the German Narcotics Act, making possession (if not medically prescribed) a criminal offense [3]. In contrast, its precursors GBL and BD, which rapidly metabolize into GHB in the body and have nearly identical effects, are legally available in many countries due to their relevance in industry [4]. GBL is often marketed as an industrial chemical, such as paint stripper or cleaning solvent, BD is an intermediate chemical product, and both GBL and BD are easily available at a low cost [5, 6]. This has contributed to their rising use as substitutes for GHB and as raw materials for the illicit home synthesis of GHB [6]. Due to their similar effects [4] and to enhance readability, we will refer to all three substances as GHB throughout.

The effects of GHB have a steep dose–response and in quality are highly dose dependent, ranging from mild euphoric effects to severe sedation and even fatal outcomes [1, 7]. At lower doses (a typical initial dose is 0.8–1.2 ml of GBL or 1–2 ml of GHB, depending on body weight, tolerance, and other factors), stimulating effects such as mood enhancement, anxiolytic effects, increased sociability, disinhibition, heightened libido, and sexual stimulation make it particularly popular in party, nightlife, and sexual (“chemsex”) contexts [8, 9]. In contrast, the substance’s sedative properties are more relevant for heavy users to mitigate withdrawal symptoms and facilitate sleep [2].

GHB carries high risks, primarily due to its narrow safety-to-harm margin, resulting in frequent accidental overdoses [1, 10, 11]. Acute GHB intoxication can result in central nervous system depression, leading to respiratory arrest, unconsciousness, and, in extreme cases, death. The risk is exacerbated when GHB is combined with other (especially GABA-ergic) substances, such as alcohol or benzodiazepines, which is a common practice in party or afterparty settings. Although the direct physical toxicity of GHB is lower than that of alcohol and other recreational drugs, frequent use poses long-term risks to physical and mental health. GHB is known to be highly addictive, with rapid development of physical and mental dependence. Especially at high levels of physical dependence, GHB withdrawal is associated with severe symptoms and is even more difficult to manage than withdrawal from alcohol, heroin or other psychoactive substances (partly due to limited clinical experience and routine in handling it). It involves symptoms such as tremors, agitation, delirium, and in severe cases, life-threatening seizures. Thus, GHB withdrawal requires medical care, often in intensive care units, due to the risk of severe complications (particularly since the less complication-prone detoxification with pharmaceutical GHB is still rarely used in Germany) [7, 12]. In Europe, GHB was the fourth most common substance involved in drug-related emergencies in hospitals in 2022, accounting for 11% of acute drug toxicity presentations and 27% of critical care admissions [13, 14]. Due to the high prevalence of GHB-associated overdoses and other medical emergencies, the substance represents a significant concern for club owners, event organizers, and ultimately policymakers [15].

There are few reliable data on the prevalence of GHB use; 10 to 20 years ago they were estimated between 0.1% and 1.3% in the general population, with considerably higher rates among partygoers [2, 5]. While GHB use was often associated with specific contexts, such as men who have sex with men (MSM), it recently appears to have spread much more broadly [2, 9]. Although overall increases in GHB use have been noted across multiple regions in Europe, North America, and Australia [2, 16–19], national and sub-national data reveal marked regional differences. For instance, while GHB use in the general population has declined in the Netherlands over recent years [20], 30-day prevalence within specific nightlife scenes (e.g., in Berlin) rose from 2% in 2015 to 9% in 2019 [19, 21]. Crises—such as the COVID-19 pandemic—seem to have partly fueled this trend [22–24].

Despite the growing trend of GHB use and its associated risks, there is a clear lack of prevention and treatment services to address this emerging issue. Reliable information on GHB is often scarcely available to users, resulting in use without adequate knowledge of its risks [3, 25]. The few existing campaigns in Germany have, in part, even been counterproductive, reinforcing the stigma associated with GHB (often mistakenly due to its perceived association with "spiking"/"knockout drops" [26]) and presumably reducing the likelihood of users seeking harm reduction measures or professional support. Traditional prevention and treatment programs often do not adequately address GHB use, focusing instead on more prevalent substances. There are almost no specialized services or self-help groups dedicated to GHB, and the sparse existing services tend to be limited to the MSM community [27]. Furthermore, the existing scientific evidence is limited mainly to GHB detoxification, withdrawal, and its pharmacological treatment [7, 12, 15], which, while important, is insufficient to address the broader challenges posed by GHB use, such as (transdisciplinary) managing its high risk potential, patterns of problematic use and dependence, the social stigma surrounding GHB, and the feasibility of harm reduction and fostering recovery.

Apart from a few reports of individuals who use GHB at home [28], there is lacking evidence on preventive approaches. Similarly, there are no empirical findings on demands and support needs related to party settings, public policies, healthcare services, or users’ social networks. While some studies have explored reasons for GHB consumption despite the high associated risks [2, 29–32], there is little to no information on users’ motivations and intentions to reduce their use or the measures they take to do so. Furthermore, users’ experiences of seeking targeted support services remain underexplored. Additionally, the perspectives of users and non-users on the consequences of GHB use have not been systematically investigated, despite the importance of such data in informing prevention and treatment strategies.

The present study addresses these research gaps using a mixed-methods design. Specifically, it examines the evaluation of preventive approaches, users’ demands directed at different domains, personal measures and reasons for decreasing use, utilization of targeted services, and perceptions of the consequences of GHB use. This study differentiates between occasional users (< weekly use) and heavy users (≥ weekly use), as previous findings suggest that the effectiveness and utility of prevention approaches may vary depending on use frequency [28]. Additionally, non-users were included to consider the important perspectives of users’ social environments and bystanders from the scene. The study applies a longitudinal design with a one-year follow-up to capture changes in use patterns and related variables over time, providing insights into the temporal dynamics of GHB-related behaviors and needs.

## Methods

### Study design

A longitudinal study design with two assessment phases was used to examine consumption patterns, perceptions, and risk mitigation strategies associated with GHB. GHB users and their social environment (i.e., individuals in contact with users of GHB, such as their peers, who did not use GHB themselves), were invited to participate. The first assessment wave (T1) was conducted from November 19th, 2022, to January 16th, 2023, followed by a subsequent assessment (T2) one year later, from November 19th, 2023, to January 16th, 2024. Both assessments were carried out online via the SoSciSurvey platform. Participants were initially recruited through non-probability convenience sampling, primarily via social media channels (Instagram, Facebook, etc.) associated with organizations within Berlin’s nightlife scene, as well as through regional prevention and counseling services. For the second assessment wave, participants who provided their email address and consent to follow-up contact in the initial questionnaire received an invitation via email. Data from the first and second assessments were linked using pseudonymized codes.

Eligibility criteria required participants to be at least 18 years of age and possess sufficient language proficiency to complete the questionnaire in either German or English. Informed written consent was obtained from all participants prior to their inclusion in the study. No financial or material incentives were provided.

The study was granted ethical approval by the Ethics Committee of Charité – Universitätsmedizin Berlin (EA4/127/22) and the study protocol was pre-registered with the German registry for clinical studies (*Deutsches Register Klinischer Studien*; https://drks.de/search/de/trial/DRKS00030608).

Only individuals who completed at least the basic questions regarding GHB consumption were included in the analysis. A total of 2,196 individuals completed (at least parts of) the first assessment wave. Of these, 910 (41.4%) were contacted for the follow-up, and 240 (26.4%) of those contacted participated in the second assessment wave.

### Assessments

Experts from relevant fields, including in- and outpatient addiction and counseling services, psychiatrists, psychologists, club operators, and consumers, were involved in conceptualization of the survey. The baseline survey included various sections covering sociodemographic data (e.g., age, gender, sexual orientation, educational status) and GHB-related aspects (e.g., consumption patterns, motivations for use, negative experiences with GHB, evaluation of prevention approaches). Questions specifically addressing details of GHB use were only posed to current users, while all participants (users and non-users) were asked to evaluate prevention approaches and related aspects. Both quantitative instruments and qualitative open-text fields were used. To ensure comparability with the baseline, the follow-up survey largely included the same items, with the addition of several questions specifically addressing changes in consumption and the utilization of specialized preventive/therapeutic services within the past year.

At both assessments, the current frequency of GHB use was assessed using an ordinal scaled item (*"never", "more than 12 months ago", "less than once a month", "1–3 times per month", "1–2 times per week", "3–5 times per week"*, and *"(almost) daily"*). Four key criteria of problematic substance use regarding GHB were screened using the established CAGE-AID questionnaire, consisting of four binary (*yes/no*) items [33].

Eight items addressed the perceived usefulness of various preventive approaches (e.g., *“educational and informational materials on GHB in party settings”*) on a 5-point Likert scale ranging from − 2 *("very negative"*) to + 2 (*"very positive"*) in users and non-users. Five open-text fields were used to explore what needs and demands GHB users direct towards different contexts, including party settings, health care, politics, personal social environments, and others. At T1 and T2, a binary item (*yes/no*) assessed whether users perceived any reasons to reduce or quit GHB use, followed by an open-text field for elaboration. Another binary item (*yes/no*) and accompanying open-text field was used to determine if they had undertaken any actions to reduce or discontinue their GHB use. The 12-month prevalence of the utilization of preventive and therapeutic services among users was assessed at T2, also using a binary item (*yes/no*) and an accompanying open-text field. On a 5-point Likert scale ranging from -2 *("very negative"*) to +2 (*"very positive"*), four items inquired how participants perceived the impact of GHB use on the physical and mental health of the user, their social environment, and party settings.

### Analyses

Descriptive statistics were used to analyze the data of the total sample and the follow-up subsample, as well as to compare three user groups based on the frequency of GHB use: non-users, occasional users, and heavy users. Pearson-Chi-Square tests with Bonferroni-Holm-correction were conducted to inferentially examine differences in nominally scaled data (e.g., binary items) between the three user groups. Due to violations of parametric assumptions, Kruskal-Wallis tests with Bonferroni-Holm adjusted *p*-values and post-hoc analyses were used to test group differences in Likert-scaled items. Non-parametric partial correlations using Spearman’s rank correlation with Bonferroni-Holm-correction were conducted to assess the relationship between having already taken steps to reduce/quit GHB use (binary variable) and the perception of the impact of GHB (ordinal variable), while controlling for use frequency (ordinal variable). All statistical analyses were performed using *IBM SPSS Statistics Version 29*; the two-tailed significance level was set at 0.05; missing data were handled with pairwise deletion.

Qualitative analysis of the open-text responses (a. user demands directed at different contexts; b. personal *measures* to reduce or cease the use of GHB; c. personal *reasons* to reduce or cease the use of GHB) followed the approach of qualitative content analysis [34–36] and was conducted using the coding software *VERBI Software MAXQDA 2024* and Excel spreadsheets. In the first stage, categories were inductively formed from the data to ensure an unbiased representation. During this process, the text material was examined line by line, with responses assigned to the most fitting category using the coding function. Once no new categories occurred – after reviewing approximately 30% of the data— the category system was finalized and subsequently revised. In a second pass, all text material was coded based on the established categories. To ensure reliability, intra- and intercoder consistency was verified through an additional independent review and cross-checking with a second coder. The results of the qualitative analyses are presented descriptively with category names, examples, and their respective frequencies.

## Results

### Sociodemographic characteristics and use of GHB

A total of 2,196 individuals participated at the first assessment wave (T1). The mean age was 29.16 years (SD = 6.75; range: 18–62). Half of the sample (50.3%) identified as male, 43.0% as female, 5.9% as diverse, and 1.8% as other (e.g., non-binary, transgender, agender). Sexual orientation was heterosexual for 46.8%, homosexual for 27.1%, bisexual for 21.1%, and other (e.g., pansexual, asexual) for 5.1%. About half (52.7%) of the participants were employed, 25.0% self-employed, 4.6% unemployed/work-seeking, 21.2% enrolled at university, and 6.5% in vocational training or in school. Around one-third of the sample (31.8%) indicated a lifetime prevalence of any mental disorder.

Out of 2,196 valid responses, 38.1% reported at T1 that they had never used GHB. A total of 14.4% indicated use more than 12 months ago, and the same number (14.4%) used it less than once a month; 20.2% reported to use GHB 1–3 times per month, 9.2% 1–2 times per week, 2.6% 3–5 times per week, and 1.0% (almost) daily.^1^ For further analysis, participants were grouped into three categories based on their frequency of use: *no current use* (“never used” and “more than 12 months ago”; *n* = 1,154, 52.6%), *occasional use* (“ < 1 × per month” and “1–3 × per month”; *n* = 760, 34.6%), and *heavy use* (“1–2 × peer week”, “3–5 × per week”, and “(almost) daily”; *n* = 282, 12.8%).

A subsample of *n* = 240 individuals participated in both the T1 and T2 assessments. Sample characteristics were mostly similar to the total sample at T1, with a larger share of non-heterosexual individuals and more frequent use of GHB. Mean age of this subsample at T1 was 31.17 years (SD = 6.96; range: 19–58); 52.5% identified as male, 40.8% as female, 5.4% as diverse, and 1.2% as other. Sexual orientation was heterosexual for 37.1%, homosexual for 32.9%, bisexual for 24.2%, and other for 5.9%. No current use increased from 42.5% (*n* = 102) at T1 to 49.6% (*n* = 119) at T2. Occasional use decreased from 42.5% (*n* = 102) at T1 to 40.0% (*n* = 96) at T2, while heavy use decreased from 15.0% (*n* = 36) at T1 to 10.4% (*n* = 25) at T2.

### Evaluation of preventive approaches by users and non-users

**Fig. 1:**
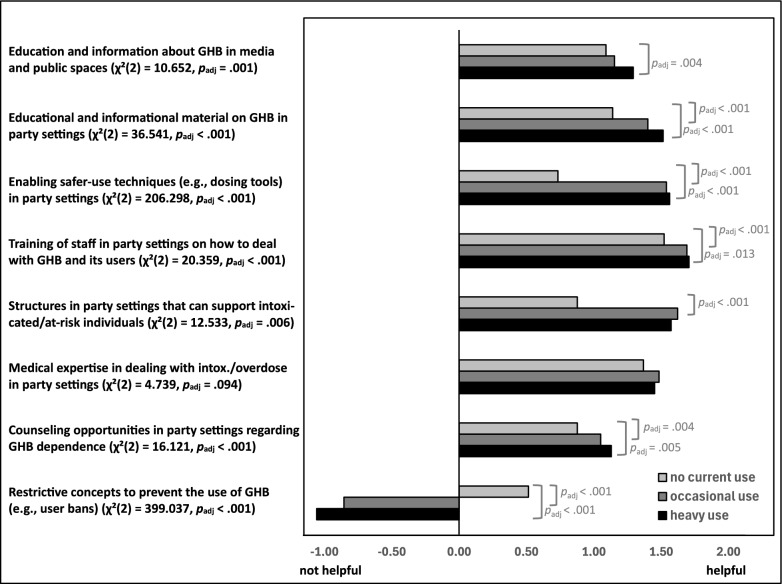
Mean ratings of the evaluation of preventive approaches clustered by user groups. *Note* User groups were clustered in no current use (“no lifetime use “ or “use  > 12 months ago “), occasional use (“ < 1 × per month” and “1–3 × per month”), and heavy use (“1–2 × per week”, “3–5 × per week”, and “(almost) daily”). The rating scale for preventive measures ranged from −2 to 2 (−2 = “negative”; −1 = “rather negative”; 0 = “neutral”; 1 = “rather positive”; 2 = “positive”). Next to the items, the results of Kruskal–Wallis tests are displayed in parentheses, while significant post-hoc pairwise comparisons with Bonferroni-Holm-correction are shown next to the respective bars

Figure 1 shows the mean ratings of various preventive approaches by user groups at T1, along with the results of inferential statistical analyses of differences in ratings between these groups at T1. Overall, except for restrictive concepts, the approaches were rated (rather) positively by all three groups. Differences are particularly noticeable between non-users compared to occasional and heavy users. All group differences were statistically significant except for the *“involvement of medical expertise in dealing with intoxicated/overdosed individuals (e.g., staff trained in first aid, paramedics in party settings)”*. The largest group difference was observed regarding “*restrictive concepts to prevent the consumption of GHB (e.g., “No-GHB-policy”, stricter door controls, banning of consumers)”*, with occasional and heavy users evaluating them negatively while non-users rated them rather positive.

To analyze change in these evaluations over time, difference scores (T2-T1) were calculated for the subsample that participated in both survey waves (*n* = 240). The median showed no change in any of the eight variables (median difference scores for all approaches were 0.00). In participants classified as heavy users at T1, approaches were rated slightly more negatively at one-year follow-up, as indicated by negative mean difference scores, while the least change was observed among non-users (*education and information in media and public spaces: M* = −0.41 vs. *M* = −0.08; *educational and informational materials in party settings: M* = −0.43 vs.* M* = 0.03; *enabling of safer-use techniques: M* = −0.50 vs.* M* = 0.06; *training of staff at party settings: M* = −0.62 vs.* M* = −0.09; *counseling in party settings: M* = −0.45 vs.* M* = 0.04; *structures in party settings: M* = −0.34 vs.* M* = 0.10; *involvement of medical expertise: M* = −0.61 vs.* M* = 0.05; *restrictive concepts: M* = −0.14 vs.* M* = 0.10).

### User demands directed at different contexts

Table 1 gives an overview of the qualitative categorization of users’ demands and suggestions (please note that qualitative categories may overlap and are intended as a schematic overview rather than strictly distinct or mutually exclusive groups). A large percentage of respondents emphasized the need for greater tolerance, destigmatization, open dialogue, and acceptance of GHB users, advocating against demonization and zero-tolerance policies, while instead promoting education, information, and safer-use practices in party settings, health care contexts, political perspectives, and personal social environments. The demand for more specialized staff and targeted support in party settings (e.g., awareness teams), as well as in health care contexts (e.g., therapeutic services), along with appropriate (state) funding for these measures, was also expressed repeatedly. Many respondents (at T1: 42.0%) perceived a need for greater knowledge among health care professionals regarding GHB use and how to manage its consequences. Regarding the political level, more than a fifth (21.7%) of respondents expressed a desire for decriminalization and legalization. Only a small percentage advocated for more restrictions and controls in party contexts (7.4%) and at the political/legal level (2.4%). Within their social environments, many participants (43.0%) called for critical reflection on substance use and a shift towards more responsible and reduced consumption. In addition, 7.2% indicated mutual support and an open dialogue within social circles as relevant.

**Table 1:** Summary of the qualitative analysis addressing the demands of users of GHB

What do you feel is lacking or what do you wish was available to you as a consumer …			
… in the party scene, particularly from the organizers (*n* = 538)			
Category title	Description of category	Examples	Percentage
Destigmatization and tolerance	Calls for reducing stigma, promoting tolerance, and replacing punitive measures with supportive approaches in party settings	“Less stigma and prejudice” “More acceptance and tolerance” “More prevention and less demonization in the party scene” “No g ban (doesn’t work) […], no drug shaming/stigma “ “No stigmatization, regardless of the drug, as this only leads to secrecy, which can result in life-threatening situations”	T1: 41.3%
Safer use and safe spaces	Calls for harm reduction by prioritizing safer use practices, providing designated areas, education, and tools at parties	“Access to dosing tools at parties” “Safer places to use” “Sufficient lighting in areas where consumption typically occurs, no stigmatization!! Safer use options instead of bans” “Safer dosing education + tools on site”	T1: 15.8%
Specialized staff and support	Calls for awareness and (specialized) support (teams) in party settings	“Awareness teams” “A team that can help without accusing or judging” “Doctors/healthcare staff, recovery rooms”	T1: 15.6%
Education and information	Calls for education, information, and open communication	“More education” “Less shaming, more information and education for everyone.” “More info about drugs. Saying ‘G is not good’ is not helpful” “To not punish, but to provide information about risk reduction strategies and knowledge on how to deal with intoxications” “More information on the risks and on safer use”	T1: 16.4%
Restrictions and control	Advocating for strict regulation, control, and repression in party settings	“A comprehensive No G policy. This drug is not good for people” “Stronger regulation” “More control and more entry bans” “Stronger body checks”	T1: 7.4%

… in health care contexts (emergency services, clinics, etc.) (*n* = 250)			
Category title	Description of category	Examples	Percentage
Destigmatization and tolerance	Calls for less stigma and judgment, and instead more acceptance and harm reduction in health care contexts	“Less stigma “ “No stigmatization of g use “ “No prejudices (regarding all drugs)“ “Less judgment “ “More understanding; safety to discuss drug use with doctors”	T1: 23.2%
Knowledge of health care professionals	Calls for more know-ledge and education about substances, harm reduction, and treatment in health care	“Knowledge about drugs” “Knowledge about the effects and treatment of intoxication” “More knowledge about harm reduction” “More awareness and knowledge of how to help people who have overdosed or are addicted” “More education for doctors and paramedics”	T1: 42.0%
Specialized services	Calls for specialized support and addiction treatment services	“Psychological/psychiatric support” “More withdrawal clinics/rehabs” “Therapy services” “Addiction counselling services”	T1: 10.0%
Providing education	Calls for education and information for the users provided by health care professionals	“Information on dosage, times and mixing” “Information about long-term effects” “Maybe flyers explaining in detail the effect and what not to mix it with” “More information and research done about the drug”	T1: 19.6%

… at political level (*n* = 249)			
Category title	Description of category	Examples	Percentage
Destigmatization and tolerance	Calls for less stigma and judgment, and instead an accepting, harm reduction-oriented policy	“Less prejudice, and judging” “Stop the drug shaming” “Less stigma, less ‘rape drug’ misconceptions” “Acceptance “	T1: 21.7%
Decriminalization and legalization	Calls for decriminalization/legalization of users and/or substances	“Legalization/decriminalization of all drugs” “Decriminalization & legalization of some substances” “Decriminalization of users”	T1: 21.7%
(Specialized) preventive and therapeutic services	Calls for specialized preventive and/or therapeutic services with appropriate funding	“More preventive measures” “State funding for awareness teams” “Drugchecking” “Addiction counselling and treatment services”	T1: 7.2%
Education and information	Calls for more know-ledge and education about GHB	“More information. The info on GHB’s effects are very limited” “More research and education” “More information about the drug, more studies about it specially when it comes to longterm use”	T1: 34.1%
Restrictions and control	Calls for strict regulations and control	“Strict bans” “More severe penalties/prosecution” “Access could be made more difficult”	T1: 2.4%

… from the social environment (*n* = 300)			
Category title	Description of category	Examples	Percentage
Destigmatization and tolerance	Calls for less stigma and judgment in (personal) social environments	“Destigmatization “ “less judgment “ “Less shaming “ “Less prejudice towards users”	T1: 33.7%
Education and information	Calls for more know-ledge and education about GHB	“More information and education “ “More knowledge “ “Provide more information, explain the risks, do not glorify G”	T1: 11.7%
Critical reflection of use; responsible/reduced use	Calls for critical reflection of substance use and a shift towards more responsible and/or reduced use	“No trivialization; conscious use” “Responsible use” “Less extreme use” “Safer drug use and more personal responsibility” “Risk awareness and consumption competence” “More active harm reduction conversations” “Less passive endorsement and idealization of substance use”	T1: 43.0%
Social support	Calls for mutual support in social environments	“Support of family/friends” “More mutual care, respect (directed towards self and others)” “Pay more attention to each other, listen to each other, express and accept criticism”	T1: 7.3%

When multiple aspects were mentioned in a response, the most relevant one was selected. Some originally German statements were translated into English for the overview. Some content overlaps between categories. A few responses did not fit into any category and were assigned to an "other" category, which is not listed in the table – thus, not all percentages add up to 100%.

### Personal measures to reduce or cease the use of GHB

More than half of the users (*n* = 512, 56.1%) indicated in the CAGE that they had have ever felt they should cut down their GHB use, amongst them almost twice as many heavy users as occasional users (68.1% vs. 35.1%; χ^2^(1) = 20.127, *p*_adj_ < 0.001). A total of 413 participants (45.3%) indicated at T1 that they had already taken measures to reduce or cease their GHB use. Significantly more heavy users had already taken measures to reduce or cease their GHB use compared to occasional users (51.8% vs. 42.9%; χ^2^(1) = 5.754, *p*_adj_ = 0.016).

The reported measures were classified into six categories through qualitative content analysis (in the following ranked by their frequency; *n* = 391 participants provided responses):

- *Reduction/cessation of use* (e.g., “using less”, “reduced dosage”, “set consumption-free days”, “stopped it during the week”, “extended abstinence intervals”, “completely cut it off”)
- *Stimulus control* (e.g., “partying less”, “change of social environment”, "avoid seeing certain friends”, “not keeping it at home", "not bringing it with me", “not buying it”)
- *Therapy and counseling* (e.g., "therapy", "addiction counseling", “addiction treatment, "detox, rehab")
- *Substitution with other substances* (e.g., "using other drugs", "choosing other drugs instead of GBL", “taking ketamine instead”, “benzodiazepines”)
- *Substitution/distraction with activities (*e.g., “sports”, “I keep myself busy, so I don’t think about it”)
- *Other measures*

### Personal reasons for reduction or cessation of GHB use

Two-thirds of participants (*n* = 589, 66.2%) indicated reasons to reduce or cease their GHB use at T1. Significantly more heavy users perceived reasons to reduce or cease their GHB use compared to occasional users (75.62% vs. 62.65%; χ^2^(1) = 13.234, *p*_adj_ < 0.001).

Table 2 presents the qualitative categorization of participants’ reasons for reducing or quitting GHB use at T1 (derived from open-text responses by *n* = 574 individuals). Physical health concerns (e.g., gastrointestinal and oral health issues) were the most frequently reported reason (19.2%), followed by immediate risks associated with GHB use, such as overdoses (15.5%). Addiction was another commonly cited reason (14.3%), while mental health consequences, including anxiety and depression, were mentioned less frequently (3.1%). Behavioral changes (e.g., disinhibited or inappropriate behavior) and personality shifts (5.6%), as well as problematic sexual behavior (2.4%), were also noted, along with impacts on social relationships (2.4%) and daily life (2.6%). Additionally, the negative social perception of GHB use was cited by some users (1.9%). In some cases, witnessing negative experiences in the social environment, instead of own experiences, contributed to the reasons for reducing use (4.4%). In other instances, the reasons were related to life changes (6.8%), such as partying less. Clearly hypothetical and not actually present reasons were categorized separately (14.8%).

**Table 2:** Summary of the qualitative analysis of the reasons to reduce or cease GHB use perceived by occasional and heavy users

Perceived reasons to reduce or quit GHB use (*n* = 574)			
Category title	Description of category	Examples	Percentage
Immediate risks / consequences	Experienced or feared severe immediate consequences of GHB use, such as overdoses, collapsing, and unconsciousness	“Overdose, unconsciousness, hospital” “Dangers overdosing” “Sometimes I had black outs” “Often going into sleepy mode” “Collapsing 3 times in a weekend” “Life-threatening risk. Besides using in clubs, I tend to use it” alone at home, which is very dangerous” “Mixing it with other substances is highly dangerous”	T1: 15.5%
Addiction and problematic use	Experiences and fears of addiction, strong cravings, frequent or high-dose use, withdrawal symptoms, and loss of control over consumption	“Addiction” “Prevent addiction” “Physical and mental dependency” “Strong cravings of taking more” “Loss of control over consumption” “Withdrawal symptoms”	T1: 14.3%
Physical health concerns	Experienced and feared physical harm from GHB, affecting various organs and systems, including digestive issues, oral health, and the immune system	“Destruction of the body” “Wounds in the oral cavity. Painful tongue. Pain while eating. Mucus in the throat. Heartburn.” “Physical (long-term) damage” “Stomach and dental health” “Immune system”	T1: 19.2%
Mental health concerns	Experiences of negative mental health impacts, including affective and cognitive effects	“High anxiety” “Makes me feel insecure mentally with extended use” “Makes depressive over time” “Depression” “I could feel it affect my memory “ “Mental health”	T1: 3.1%
Behavioral and personality changes	Short- and long-term personality and behavioral shifts, including increased disinhibition, heightened egoism, and frequent regret over actions taken	“Changes in personality” “Subtle changes in character. Even when you haven’t taken anything in a while, you feel like a different person” “Loss of sensitivity, less intelligent, loss of inhibitions, personal boundaries aren’t realized anymore; I often feel ashamed the next day about things I said (I feel alienated from myself when using too much G)” “My girlfriend says I change, and I don’t like that” “Guilt over actions taken under the influence of G” “I felt it turned me more aggressive when dealing with conflicts in a party environment” “Experienced personality shift to something I am not at all during long sessions”	T1: 5.6%
Sexual behavior	Problematic patterns in sexual behavior related to GHB use, e.g., reliance on its enhancing effects and regretted encounters	“I don’t want to depend on sex-enhancing drugs to have sex” “Wrong sexual behavior” “Random sex” “Sex with people I don’t like/find attractive afterward” “Avoid chemsex addiction”	T1: 2.4%
Social relationships and impact on others	Detrimental effects of GHB use on close social relationships, e.g., partners, friends, and family	“Spiraling into addiction, messing up relationships and friendships” “Disrespectful behavior toward friends” “Damage I’ve caused in my most important friendships, as they were very worried” “Trying not to jeopardize the partnership” “Family”	T1: 5.4%
Impact on daily life and responsibilities	Negative impacts on profession-related activities and everyday life	“GBL/GHB enables or tempts very long parties like no other drug, negatively affecting everyday performance” “Like all drugs, it threatens my professional career” “Can’t manage daily life anymore and take ages to do everything” “Consumption dictates daily life”	T1: 2.6%
Deterrent experiences in the social environment	Witnessing negative effects of GHB on individuals in the social environment; including addiction, life-threatening situations, and severe behavioral changes	“Friends who let the drug ruin their lives and drifted away despite attempts to help” “Some friends of mine are addicted” “Seen friends become more and more dependent” “So many friends died or almost died” “My ex-husband became heavily addicted to GBL and put his life at risk” “I have seen many people behaving like monsters because of it”	T1: 4.4%
Stigma and social perception	Concerns about stigma, social pressure, and negative social associations with GHB use	“Stigma” “Not wanting to be part of ‘this’. Many bad things regarding G that I don’t agree with, socially, sexually, etc., not wanting to be associated with that” “Dealing with the shame or guilt of taking it can be stressful” “Pressure in the scene, negative opinions from friends and clubs”	T1: 1.9%
Changed life circumstances and aims	Perceived changes in life circumstances that are no longer compatible with GHB use, or decision to abstain/reduce use	“Growing out of the party phase / friends no longer use it” “Travelling” “Exams / taking a break from partying” “Pregnancy” “Want to go sober again”	T1: 6.8%

When multiple aspects were mentioned in a response, the most relevant one was selected. Some originally German statements were translated into English for the overview. Some content overlaps between categories. For individuals who reported clearly hypothetical and not actually existing reasons, a separate category, "hypothetical reasons," was created (e.g., “If I would use it in daily life”, “In case of direct physical damages”), which is not listed in the Table (14.8%). A few responses did not fit into any category and were assigned to an "other" category, which is not listed in the Table (4.0%). Thus, the percentages in the table do not add up to 100%

### Utilization of preventive and therapeutic services among users

Only *n* = 4 individuals (3.6%) from the follow-up sample reported having used any preventive or therapeutic services related to their GHB use in the past 12 months. The services they accessed included addiction counseling, educational information evenings, psychotherapy, drug counseling, withdrawal programs, and drug rehab.

### Perceptions of the consequences of GHB use

**Fig. 2:**
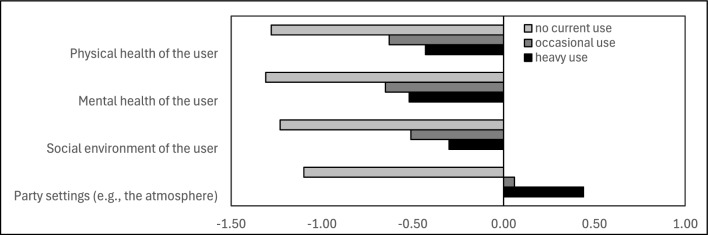
Mean ratings of the consequences of GHB use (the impact on the physical and mental health of the user, on its social environment, and on party settings) differentiated by user groups. *Note* User groups were clustered in no current use (“no lifetime use “ or “use > 12 months ago “), occasional use (“ < 1 × per month” and “1–3 × per month”), and heavy use (“1–2 × per week”, “3–5 × per week”, and “(almost) daily”). The rating scale for the consequences ranged from −2 to 2 (−2 = “negative”; -1 = “rather negative”; 0 = “neutral”; 1 = “rather positive”; 2 = “positive”)

Figure 2 shows the mean ratings of the impact of GHB use at T1—on the physical and mental health of the user, their social environment, and on party settings—differentiated by user groups. Regarding all four target areas, there were significant group differences (physical health: χ^2^(2) = 296.799, *p*_adj_ < 0.001; mental health: χ^2^(2) = 286.462, *p*_adj_ < 0.001; social environment: χ^2^(2) = 306.377, *p*_adj_ < 0.001; party settings: χ^2^(2) = 496.507, *p*_adj_ < 0.001). Non-users rated the impacts most negatively, followed by occasional users (all differences between non- vs. occasional and non- vs. heavy users were significant in the post-hoc analyses with Bonferroni-Holm-correction; the difference between occasional vs. heavy users only was significant for the impact on physical health and party settings).

Statistically controlling for use frequency, non-parametric partial correlations at T1 revealed that the probability that users had already initiated measures to reduce their use was higher the more negatively they rated the impact of GHB on the physical health of the users (*r*_*S*_ = 0.145, *p*_adj_ < 0.001), the mental health of the users (*r*_*S*_ = 0.199, *p*_adj_ < 0.001), their social environment (*r*_*S*_ = 0.206, *p*_adj_ < 0.001), and party settings (*r*_*S*_ = 0.165, *p*_adj_ < 0.001).

To assess the variability of these perceptions over time, difference scores (T2-T1) were calculated for the subsample that participated in both assessment waves (*n* = 240). The median showed no change in any of the four variables (the median difference scores for all four areas of impact were 0.00), and the arithmetic means indicated only little change as well (physical health: *M* = −0.03; mental health: *M* = −0.08; social environment: *M* = −0.13; party settings: *M* = -0.15).

## Discussion

This study investigated the evaluation of preventive approaches, users’ demands directed at different domains, personal measures and reasons for decreasing use, utilization of preventive and therapeutic services, and perceptions of the consequences of GHB use among occasional and heavy GHB users and their social environment.

The overall positive evaluation of most proposed preventive approaches (e.g., educational strategies and supportive structures in party settings) by users and their social environments suggests that these strategies would likely be well-accepted and potentially effective. With the exception of restrictive approaches, occasional and heavy users rated the proposed strategies more positively than non-users, likely because their direct experiences with GHB-related risks make them more receptive to harm reduction measures that address their specific needs and are viewed as supportive rather than punitive and stigmatizing. These findings are in line with the assumptions of harm reduction frameworks (e.g., [38, 39]), indicating that such harm reduction interventions are likely to be particularly well-received by the target group and should be prioritized over restrictive approaches. The mostly stable evaluation of these approaches by users and non-users over time further supports their potential for long-term effectiveness in reducing GHB-related harm.

The qualitative analysis of open-text responses on aspects missing from prevention and treatment revealed several major gaps and issues. Notably, the demands and needs expressed by users across different contexts—such as party and health care settings, politics, and personal social environments—were strikingly similar. Many users expressed a desire for greater tolerance, destigmatization, and an open dialogue about GHB use in all these contexts. There were also widespread calls for more education and harm-reduction strategies (e.g., safer-use practices) rather than demonization and restrictive approaches. Additionally, the demand for greater knowledge about GHB among health care professionals was particularly pronounced. These findings highlight the substantial and multifaceted need for adequate supportive strategies. They also suggest that addressing the risks of GHB use effectively and sustainably requires a comprehensive approach, integrating medical, social, and political perspectives.

Many participants called for critical reflection on substance use and a shift toward more responsible use within their social environments. This suggests that harmreduction strategies could encompass promoting non-stigmatizing self-reflection on use patterns. Established strategies from other substance use contexts, such as consumption diaries, could serve as effective tools to facilitate these reflective processes [40].

A significant proportion of GHB users, particularly heavy users, indicated reasons to reduce or quit their use, with physical health concerns, immediate risks associated with GHB, and addiction reported most frequently. This suggests a clear window of opportunity for intervention and highlights key areas that should be considered in preventive and treatment strategies. These findings also demonstrate the users’ abilities to reflect on negative aspects of their consumption, suggesting that strategies should be tailored to address and build upon these. Alongside the reasons for decreasing use, understanding the reasons for initiation and maintenance of GHB use is also important to inform prevention and treatment efforts [2, 29, 31, 32] and should be considered in future investigations.

Behavioral changes, and especially personality changes, due to (prolonged) GHB use were repeatedly reported. This highlights a specific aspect of GHB that is not commonly seen in this intensity with other substances and should be considered in the targeted conceptualization of preventive and therapeutic measures. The numerous, sometimes very disturbing experiences in the social environment, such as the deaths of friends caused by GHB use, further emphasize the high risks associated with GHB and underscore the importance of implementing appropriate harm reduction strategies.

Despite a large proportion of participants indicating a desire to reduce or quit GHB use, only a small number accessed preventive or therapeutic services within the last year. This reveals a substantial gap between the desire for change and the accessibility or use of support services. The qualitative categorization of measures taken by users to reduce or cease GHB use further highlights a reliance on personal efforts, such as reducing dosage or frequency or engaging in stimulus control, rather than seeking professional help. These findings further emphasize the demand for more accessible, low-threshold, non-stigmatizing support strategies. At the same time, they also underscore users’ self-efficacy and conscientiousness. Many reported successfully adjusting their consumption patterns to make them less harmful. These personal resources should be considered when designing strategies. Educationally strengthening these self-management strategies, alongside offering more comprehensive professional support when needed, may be an effective approach.

Non-users rated the impact of GHB on physical and mental health of the users, their social environment, and party settings more negatively than occasional users and, particularly, heavy users. This disparity could be attributed to several factors: For one, non-users may have less familiarity with the substance and thus perceive its risks as more severe, possibly influenced by external sources such as media or secondhand bad experiences. In contrast, heavy users might downplay the negative consequences, due to desensitization, normalization, or cognitive dissonance [41–45]. Desensitization reduces the perceived severity of risks through repeated exposure – for instance, frequent experiences with overdoses may make such events feel less shocking. Normalization within peer groups further contributes by framing substance use as routine or acceptable. Cognitive dissonance reinforces this pattern by leading users to rationalize and justify their behavior, minimizing perceived harms to align their beliefs with continued use. This discrepancy becomes particularly evident when considering that heavy users, while downplaying negative impacts of GHB, most frequently reported personal reasons for why they should reduce their GHB use. Occasional users may fall in between, acknowledging some risks but not to the same extent as non-users. This perception gap has significant implications for prevention strategies, suggesting that educational campaigns should adequately address the downplaying and normalization of GHB use among (heavy) users. This is further underlined by the finding that the more negatively users perceived the impact of GHB, the more likely they were to have taken steps to reduce their use. Interventions targeting non-users should instead focus on providing accurate information to prevent misperceptions which could reinforce stigma. Tailored strategies for each group could increase the effectiveness of prevention and harm reduction efforts.

The findings also highlight the ongoing need to balance harm reduction vs. deterrence and restrictive strategies, requiring careful consideration of the target audience and the specific objectives [46, 47]. Deterrence strategies, which emphasize risks and negative consequences, often leveraging fear or stigma, can be particularly effective in preventing individuals—especially non-users—from initiating substance use. In contrast, harm reduction strategies are central to secondary prevention by focusing on minimizing the damage of substance use. This approach recognizes that abstinence may not be desirable, achievable or realistic for all users and instead prioritizes practical support to reduce risks, such as overdoses or long-term complications.

Only a relatively small proportion of participants (*n* = 240; including only *n* = 36 (T1) / *n* = 25 (T2) heavy users) completed both assessments, and within this group, only minimal changes in use frequency were observed. This raises the question of selection effects influencing the composition of the follow-up group. This group may overrepresent individuals who are particularly stable in their use behaviors or those more motivated to participate in longitudinal research. Conversely, individuals who did not complete the follow-up measurement might include those with more chaotic or escalating use patterns, or those who disengaged from the study for other reasons. The limited number of participants who reduced their GHB use during the follow-up period may also reflect that significant behavior change within this population is challenging. This may be partially attributable to psychological and social reinforcement processes associated with GHB use.

### Strengths and limitations

Our study was the first to empirically investigate these research questions, utilizing a mixed-methods approach that includes both quantitative and qualitative data. The qualitative component allowed for an unbiased and comprehensive collection of information, providing deeper insights into the experiences of users, while the quantitative component facilitated the identification of patterns and relationships. The study was informed by expert input during its conceptualization, and benefits from a large sample size and the inclusion of heavy users, who are often underrepresented in research. Including non-users also enabled the integration of various perspectives. With a smaller subsample that participated in the follow-up, our study is the first with a longitudinal design to examine changes in GHB use and related aspects over time.

However, there are limitations to consider. The convenience sample may not be fully representative of the underlying population, potentially introducing selection bias. The reliance on self-reported data also poses the risk of inaccurate or biased responses. Forming qualitative categories proved challenging, with some overlap between categories, potentially limiting the clarity of findings. In addition, the question on reasons for reducing or quitting GHB use was not clearly worded, leading to ambiguity as to whether they were hypothetical vs. existing reasons. Furthermore, the survey had to be kept concise to avoid overwhelming participants, which may have constrained the depth of information gathered. Additionally, all findings are based on observational data, which does not allow for causal conclusions and may be influenced by unconsidered confounding variables (such as participants’ mental health status, physical health conditions, substance use history, socioeconomic factors, or other lifestyle-related variables). Ideally, future studies should build on these findings and test the impact of specific prevention and treatment approaches through randomized controlled designs.

## Conclusions

In summary, our findings highlight the necessity of developing and implementing more comprehensive and inclusive prevention and treatment strategies to reduce the complex risks associated with GHB use. By focusing on harm reduction strategies, increasing access to support services, and addressing the stigma associated with GHB use, interventions are likely to better meet the needs of this growing population. At the same time, the findings emphasize a sense of responsibility among users, which should be reinforced and integrated into adequate approaches. Given the pronounced risks associated with GHB, particularly among heavy users, immediate action is required through an interdisciplinary effort involving policymakers, health care professionals, and the society to close the gaps in prevention and treatment. Our findings can serve as an important foundation for these efforts.

## Data Availability

The dataset used and analysed during the current study is available from the corresponding author on reasonable request.

## Abbreviations

BD: 1,4-Butanediol (also known as 1,4-BD or BDO; precursor substance of GHB)
COVID-19: Coronavirus disease 2019
EMCDDA: European monitoring centre for drugs and drug addiction
EUDA: European Union Drugs Agency
GBL: Gamma-butyrolactone (precursor substance of GHB)
GHB: Gamma-hydroxybutyrate / 4-Hydroxybutanoic Acid / γ-Hydroxybutyric Acid
MSM: Men who have sex with men
T1: First assessment wave (11/2022–01/2023)
T2: Second assessment wave (11/2023–01/2024)
UNODC: United Nations Office on Drugs and Crime
WHO: World Health Organization

## Footnotes

1 A nuanced analysis of consumption frequencies by sociodemographic characteristics and other relevant variables is available in the article by [37].
